# The GSK-3 Inhibitor CT99021 Enhances the Acquisition of Spatial Learning and the Accuracy of Spatial Memory

**DOI:** 10.3389/fnmol.2021.804130

**Published:** 2022-01-27

**Authors:** Yeseul Lee, Zuner A. Bortolotto, Clarrisa A. Bradley, Thomas M. Sanderson, Min Zhuo, Bong-Kiun Kaang, Graham L. Collingridge

**Affiliations:** ^1^Department of Brain and Cognitive Sciences, College of Natural Sciences, Seoul National University, Seoul, South Korea; ^2^Centre for Synaptic Plasticity, School of Physiology, Pharmacology and Neuroscience, University of Bristol, Bristol, United Kingdom; ^3^Lunenfeld-Tanenbaum Research Institute, Mount Sinai Hospital, Toronto, ON, Canada; ^4^Neurosciences and Mental Health, The Hospital for Sick Children, Toronto, ON, Canada; ^5^Genes and Genome Biology, The Hospital for Sick Children, Toronto, ON, Canada; ^6^Department of Physiology, University of Toronto, Toronto, ON, Canada; ^7^Department of Biological Sciences, College of Natural Sciences, Seoul National University, Seoul, South Korea; ^8^Tanz Centre for Research in Neurodegenerative Diseases, Toronto, ON, Canada

**Keywords:** GSK-3, NMDAR-dependent LTD, spatial learning and memory, CT99021, Morris water maze, DMTP T-maze, memory enhancement

## Abstract

Glycogen synthase kinase 3 (GSK-3) is a Ser/Thr protein kinase that regulates many cellular processes, including synaptic plasticity. Previously, we reported that inhibition of GSK-3 prevents the induction of one of the major forms of synaptic plasticity, N-methyl-D-aspartate receptor (NMDAR)-dependent long-term depression (LTD), in hippocampal slices. In the present study, we have investigated the effects of inhibiting GSK-3 on learning and memory in healthy naïve animals. Systemic administration of a highly selective GSK-3 inhibitor, CT99021, reversibly blocked NMDAR-dependent LTD in the CA1 region of the hippocampus in anesthetized adult mice. In behavioral tasks, CT99021 had no effect on locomotor activity, anxiety, hippocampus-dependent contextual fear memory, and hippocampus-dependent reversal learning. However, CT99021 facilitated the rate of learning in the Morris water maze (MWM) and T-maze and enhanced the accuracy of long-term spatial memory in the MWM. These findings suggest that GSK-3 regulates the accuracy of spatial memory acquisition and recall.

## Introduction

GSK-3 is a Ser/Thr kinase that is involved in multiple signaling pathways including glucose regulation (Embi et al., 1980; Parker et al., 1983), cell proliferation and migration (Jope et al., 2007; Beurel et al., 2010) and inflammation and apoptosis (Jope et al., 2007, 2017; Beurel et al., 2010). In mammals, GSK-3 has two paralogs, GSK-3α and GSK-3β, which are activated by de-phosphorylation at Ser21 and Ser9, respectively. GSK-3 is highly expressed in the Central Nervous System (CNS; Lau et al., 1999; Leroy and Brion, 1999) and has been implicated in the pathology of many brain disorders, including Alzheimer’s disease (AD; Pei et al., 1997, 1999; Cohen and Goedert, 2004; Hooper et al., 2008), bipolar disorder (Beasley et al., 2002; Muneer, 2017), schizophrenia (Beasley et al., 2002; Kozlovsky et al., 2002; Emamian, 2012), fragile X syndrome (Mines et al., 2010; Mines and Jope, 2011; Franklin et al., 2014), and Down’s syndrome (Trazzi et al., 2014). For example, increased levels of hippocampal GSK-3 expression are observed in human AD patients (Pei et al., 1997; Blalock et al., 2004). In AD, GSK-3 facilitates the production of amyloid beta (Aβ), the main element of amyloid plaques (Phiel et al., 2003), and hyper-phosphorylates tau, resulting in neurofibrillary tangles. Importantly, behavioral deficits in various AD models can be rescued by either GSK-3 inhibitors (Rockenstein et al., 2007; Hu et al., 2009; Ding et al., 2010; Onishi et al., 2011; Ly et al., 2013; Griebel et al., 2019) or by knockdown of GSK-3 paralogs (Hurtado et al., 2012). Furthermore, the learning deficits caused by over-expression of GSK-3 are ameliorated in Tau-knockout mice (Gomez de Barreda et al., 2010). Collectively, these observations strongly implicate altered GSK-3 activity in the etiology of AD (Cohen and Goedert, 2004; Hooper et al., 2008; Kremer et al., 2011; Llorens-Martin et al., 2014). Given the involvement of GSK-3 in AD and other major brain disorders it is important to understand its normal functions in the CNS.

The use of genetically modified mice has implicated GSK-3 in a variety of higher cognitive functions, such as spatial learning and memory (Hernandez et al., 2002; Liu et al., 2003), novel object recognition memory (Engel et al., 2006; Pardo et al., 2015) and social behaviors (Khlghatyan and Beaulieu, 2020). In terms of pharmacology, GSK-3 inhibitors lead to enhanced memory in the passive avoidance task (Yuskaitis et al., 2010), cued fear conditioning (Watase et al., 2007; Lipina et al., 2013), spatial learning (Nguyen et al., 2017) and object recognition memory (Lipina et al., 2013). The mechanism that likely underlies GSK-3’s role in these cognitive processes is altered synaptic plasticity (Bradley et al., 2012). Initial studies showed that in the hippocampus two types of NMDAR-dependent synaptic plasticity, long-term potentiation (LTP) and LTD, are regulated by GSK-3. Specifically, the over-expression of GSK-3β inhibits LTP (Hooper et al., 2007) and the pharmacological inhibition of GSK-3 inhibits LTD (Peineau et al., 2007). Inhibition of LTD was observed with a variety of GSK-3 inhibitors, but not other Ser/Thr kinase inhibitors, such that off-target effects of kinase inhibitors could be discounted. This effect was highly specific for NMDAR-LTD since the same inhibitors had no effect on basal synaptic transmission, LTP, depotentiation or metabotropic glutamate receptor (mGluR-) LTD. Considerable evidence has since accumulated suggesting that GSK-3 is a pivotal kinase regulating the balance between LTP and LTD (Dewachter et al., 2009; Bradley et al., 2012; Besing et al., 2017; Draffin et al., 2021).

In the present study, we used a highly selective (Bain et al., 2007) and systemically active (Pan et al., 2011) GSK-3 inhibitor, CT99021 (also known as CHIR99021), to investigate the role of GSK-3 in LTD *in vivo* and to further explore the function of GSK-3 in cognitive processes. We confirmed that CT99021 is able to affect brain function when applied systemically. We found that it was able to inhibit, in a reversible manner, NMDAR-LTD in adult anesthetized mice. In behavioral tests, CT99021 did not affect gross motor skills, anxiety or fear conditioning. Surprisingly, it also had no effect on reversal learning in a hippocampus-dependent task. It did, however, increase the rate of learning and improved target accuracy in a spatial memory task in the MWM and accelerated learning in a T-maze. These data suggest that GSK-3 may be active during exploration and that its inhibition improves the rate of spatial learning and the accuracy of spatial memory.

## Materials and Methods

### Animals

All experiments were performed using 9–11 week-old mice. Male C57BL/6J mice were used for behavioral experiments (Jackson Laboratories). Mice were housed in batches of 3–4 per cage and food and water were provided at all times. The animal facility was kept under a 12 h light/dark cycle with set temperature and humidity controls. All behavioral experiments were conducted under the policy for the care and use of laboratory animals approved by the Animal Care and Use Committee of Seoul National University. Electrophysiology experiments were performed using C57BL/6N mice (Charles River) of both sexes in compliance with the UK Animal Scientific Procedure Act 1986.

### Compounds

The GSK-3 inhibitor CT99021 was purchased from Cayman and Tocris (Cayman: cat# 13122; Tocris: cat# 4423). The drug was firstly dissolved in 100% DMSO and was then diluted in a mixture of polyethylene glycol 400 (Fisher) and saline solution (0.9% NaCl). The final solution consisted of 10% DMSO, 45% Polyethylene glycol 400 and 45% saline. The solution containing CT99021 was administrated by intraperitoneal injection (i.p.) at 25 mg/kg. Drug administration took place every day 1 h prior to the behavior tests apart from delayed match-to-place T-maze (DMTP T-maze). For the DMTP T-maze, drug injection was carried out 30 min prior to the test. The experimenter was blinded to whether the mouse received CT99021 or vehicle. Ketamine (100 mg/ml) was diluted to 1 mg/ml in saline solution and injected (i.p.) at 20 mg/kg.

### Forced Swim Test

The forced swim test (FST) was conducted in a glass cylinder filled with warm water and maintained at 25 ± 1°C. The test consisted of a single 6 min session. Immobile time during the last 4 min was measured by Ethovision (Noldus) and used for analysis (Pan et al., 2011).

### Open Field Test

The open field test (OFT) was conducted in an opaque box made of white acrylic (40 × 40 × 40 cm) under a dim light for 10 min allowing mice to move freely. Movement was recorded by a ceiling mounted camera. An inner square zone (20 × 20 cm) was designated as the center and the remainder outer rim as the edge. Locomotor activity was assessed by measuring the total distance (cm). The basal anxiety level of mice in the OFT was assessed by determining the percentage of time spent in the two zones.

### Elevated Plus Maze

An elevated plus maze (EPM), consisting of four arms (two open arms and two closed arms), was installed 58 cm above the ground. Each mouse was placed at the center of the maze facing an open arm and movement was recorded for 5 min. The time spent in open arms or closed arms was calculated by an automated animal tracking program (Ethovision 3.1, Noldus). The time spent in the center of the maze is not presented.

### Contextual Fear Conditioning

All mice were handled for 4 consecutive days prior to the contextual fear conditioning (CFC). On the conditioning day, mice were exposed to a Coulbourn chamber (Coulbourn, H10-24T) for 2 min and 28 s allowing them to explore the area before a 2 s-foot shock (0.2 mA or 0.6 mA) was given. Mice were removed from the chamber 30 s after the foot shock. On the following day (retrieval day), mice were re-exposed to the same chamber, without the foot shock, for 3 min and their freezing time was recorded by a camera installed inside of the chamber. Freezing behavior was analyzed with the program, Freeze Frame (Coulbourn).

### Morris Water Maze

An opaque gray tank (70 cm in radius, 100 cm height) was used for the MWM. The hidden platform (5 cm in radius) was placed in the tank and submerged with water (1 cm below the surface). White colored paint was dissolved in the water to obscure the platform. Experiments were conducted under a dim light and 4 visual cues were present around the tank. Water temperature was maintained at 21 ± 1°C. Animal movements were recorded by a ceiling mounted camera and analyzed using Ethovision 3.1 (Noldus). After 4 handling days, 5 days of training were conducted. On training days, mice were placed in the tank and allowed to search for the hidden platform for 60 s. If they were unable to find the platform in the time, animals were guided to the platform and given 10 s to remember spatial cues before being returned to the home cage. Every animal was tested 4 times a day with a 1 min interval between tests and presented to the MWM from different starting points chosen in a randomized order. After 5 days of training, a probe trial was conducted (Day 6, probe 1). For this, the hidden platform was removed and mice were introduced to the tank and allowed to swim freely for 60 s. For analysis, the arena was divided into 4 quadrants, with the one in which the hidden platform was located being designated as the target quadrant. Time spent in the vicinity of the platform was defined as time spent within a vicinity zone (10 cm in radius) centered on the platform location. For reversal training (Day 7 and 8), the platform was placed in the opposite side to the target quadrant. For probe trial 2 (Day 9, probe 2), the platform was removed again and mice were tested as in probe trial 1. Gallagher’s proximity measure was uncorrected for entry to the maze or swim speed. Heat maps were generated using Ethovision Heatmap generator (Noldus information technology).

### Delayed Match-to-Place T-Maze

To increase reward seeking behavior, all mice underwent food deprivation a week prior to the initiation of the test until completion of the test. The target weight was set to 85% of their original weight. Food was individually provided (1.5 g pellet/24 g) after the test each day. 70 μl of condensed milk diluted at 1 : 1 ratio with distilled water was provided as a reward. Before the initiation of the test, animals were habituated in the T-maze for 10 min for 2 days. During the habituation period, animals were presented with a reward in both of the open arms of the T-maze. The reward was refilled every time animals consumed one to encourage exploratory behavior. Training consisted of two sessions; forced choice and free choice. During the forced choice session, only one arm was open and carried a reward. Animals were allowed to consume and explore the arm for 90 s before being placed back into the starting arm. In the free choice session, conducted after a 10 s delay, animals were given 90 s to choose either of the two open arms, but only the previously blocked arm carried a reward. When animals entered the previous blocked arm with a reward in the free choice session, it was marked as correct choice. After 5 days of training, the open arm during the forced choice session was switched for reversal learning and animals were tested for an additional 3 days. Animals had four trials a day with a minimum of a 15 min interval between trials. Correct choice (%) was calculated daily and used for analysis.

### Electrophysiology

C57BL/6N mice were anesthetized with pentobarbital (60 mg/kg, i.p.). The stimulating electrode was placed in the Schaffer collateral-commissural (SCC) pathway in area CA1 [anterior-posterior (AP): -1.9, mediolateral (ML): -2.0, depth (D): -1.3] and the recording electrode was positioned at the ipsilateral stratum radiatum (AP: 2.0, ML: -1.4, D: -1.2). A baseline of synaptic responses, evoked at a frequency of 0.03 Hz, was obtained for 30 min prior to the administration of CT99021 (25 mg/kg, i.p.) or ketamine (20 mg/kg, i.p.). Low frequency stimulation (LFS; 1 Hz, 900 pulses) was given 15 min later for ketamine and 60 min later for CT99021 in an attempt to induce LTD. In each experiment, LFS was delivered firstly in the presence of a drug and then 90 min later, to test for the reversibility of any effect. Field EPSPs were acquired and analyzed using WinLTP (Anderson and Collingridge, 2007). At the end of each experiment, high-intensity current was given through stimulating and recording electrodes, resulting in a lesion at the tip of the electrodes, in order to confirm their placements. The animal was decapitated and the brain was removed. Parasagittal slices (250∼300 μm thick) were prepared in ice cold 0.9% NaCl solution and images of the lesions were taken. Experiments in which the electrodes placements were incorrect were excluded from analysis.

### Statistics

Results are presented as mean ± standard error of the mean (SEM). For statistical comparisons, unpaired *t*-tests, repeated measures ANOVA, one-way ANOVA or two-way ANOVA with Sidak’s *post hoc* test were used as appropriate. ^***^p < 0.001, ^**^p < 0.01, *p < 0.05.

## Results

### CT99021 Blocks the Induction of NMDAR-LTD in Adult Anesthetized Mice

**FIGURE 1:**
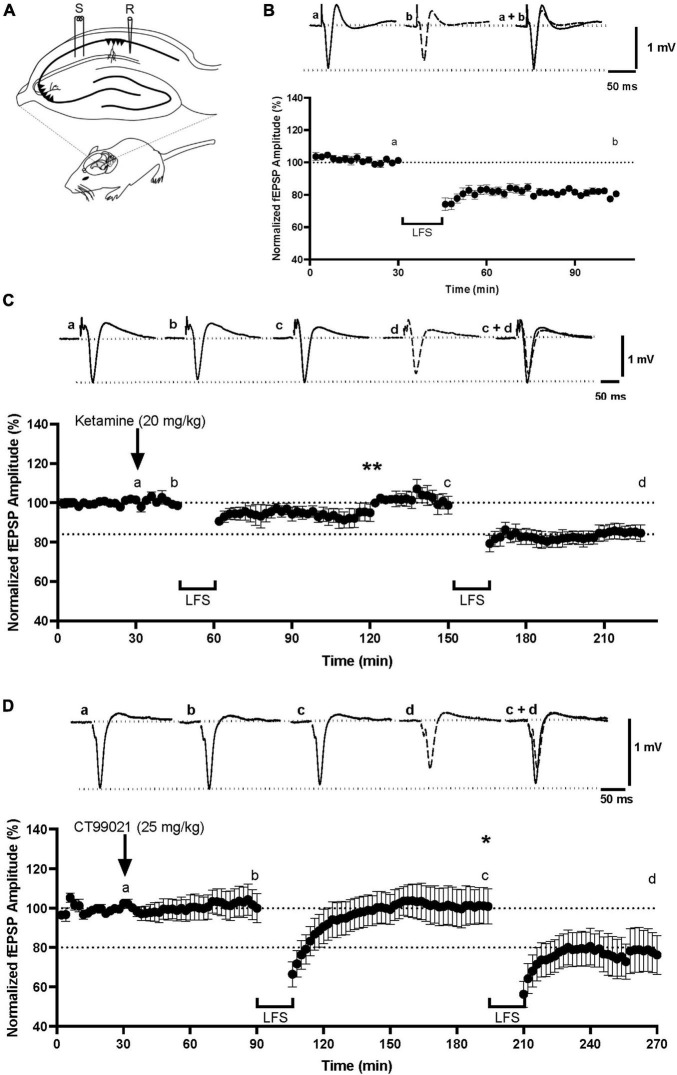
CT99021 reversibly blocks the induction of NMDAR-LTD at CA1 synapses *in vivo*. **(A)** Schematic drawing of electrode placements in anesthetized adult mice (S: stimulating electrode, R: recording electrode). **(B)** NMDAR-LTD in anesthetized mice using LFS (*n* = 7). **(C)** Ketamine, 20 mg/kg (i.p.), blocks the induction of LTD (**p < 0.01, one-way ANOVA) in a reversible manner (*n* = 4). **(D)** CT99021, 25 mg/kg (i.p.), blocks NMDAR-LTD (*p < 0.05, one-way ANOVA) in a reversible manner (*n* = 3). The inserts are averages of four successive traces taken from the experiments at the time indicated on the plots. Data are presented as mean ± SEM.

Previous studies have shown that LTD can be induced in area CA1 of adult anesthetized mice (Kimura et al., 2014; Figure 1A) using a train of low frequency stimulation (LFS; 900 shocks, 1 Hz). Consistent with earlier observations, we found that LFS induced a moderate, but robust LTD (80.5 ± 1.2% of baseline, measured at 104 min; Figure 1B) that was not observed when LFS was applied 15 min after injection of the non-competitive NMDAR antagonist, ketamine, at 20 mg/kg i.p. (95.0 ± 4.3% measured at 120 min; *p* < 0.01, one-way ANOVA, LTD under control conditions vs. in the presence of ketamine; Figure 1C). When a second LFS was given 120 min after the ketamine injection, a time when it would be expected to cleared from the brain (Moghaddam et al., 1997), LTD that was similar to the control LTD was observed of 84.6 ± 4.3% (*p* > 0.05, one-way ANOVA, LTD under control conditions vs. LTD after washout of ketamine; Figure 1C). This demonstrates that LFS can induce NMDAR-LTD in anesthetized mice under our experimental conditions.

We next tested whether inhibition of GSK-3 activity can block the induction of NMDAR-LTD *in vivo* using a dose (25 mg/kg, i.p.) previously determined to be effective in behavioral experiments (Pan et al., 2011). CT99021 was injected 1 h prior to the delivery of LFS. In all cases, there was a transient depression of synaptic transmission following LFS, but no LTD (101.0 ± 8.9% measured at 194 min; *p* < 0.05, one-way ANOVA, LTD under control conditions vs. in the presence of CT99021; Figure 1D). The effect of CT99021 was fully reversible, since a second LFS, delivered 2 h 45 min after the CT99021 injection, when the effects should have subsided (Pan et al., 2011), resulted in LTD (76 ± 10% measured at 270 min) comparable to the control LTD (*p* > 0.05, one-way ANOVA, LTD under control conditions vs. LTD after washout of CT99021; Figure 1D). Therefore, the induction of NMDAR-LTD was blocked in a reversible manner by CT99021, administered i.p. at a concentration of 25 mg/kg. This confirms that CT99021 engages targets in the hippocampus *in vivo* and that our protocol is suitable for testing the effects of this drug on behavior.

### Basal Anxiety Level and Locomotor Activity Is Not Affected by CT99021

**FIGURE 2:**
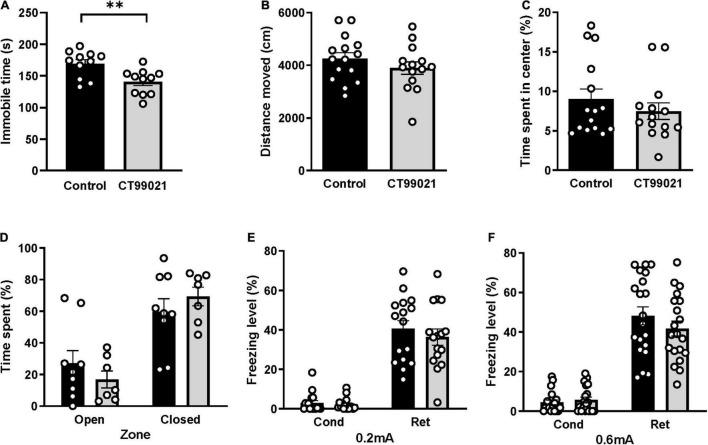
CT99021 does not affect locomotor activity, basal anxiety levels or contextual fear memory. **(A)** Immobile time (s) measured in the FST 1 h after vehicle (*n* = 11) or CT99021 (*n* = 11) injection (^**^p < 0.01, unpaired *t*-test). **(B)** Total distance moved (cm) for control (*n* = 15) and CT99021-treated (*n* = 14) mice in the OFT (*p* > 0.05, unpaired *t*-test). **(C)** Time spent (%) in the central region of the arena for these same groups of mice in the OFT (*p* > 0.05, unpaired *t*-test). **(D)** Time spent (%) for control (*n* = 9) and CT99021-treated (*n* = 7) mice in the open and closed arms of the EPM (*p* > 0.05, two-way ANOVA). **(E)** Freezing level (%) in the contextual fear conditioning test for control (*n* = 16) and CT99021 (*n* = 16) groups using a 0.2 mA foot shock (*p* > 0.05, two-way ANOVA). **(F)** Freezing level (%) of control (*n* = 20) and CT99021 (*n* = 20) groups using a 0.6 mA foot shock (*p* > 0.05, two-way ANOVA). Cond: conditioning day, Ret: retrieval day. Control: vehicle group (black histograms), CT99021: drug treatment group (gray histograms). Data are presented as mean ± SEM.

Messenger RNA expression of GSK-3 is tightly correlated with depressive-like behavior in the FST (Pavlov et al., 2017), and CT99021 treatment attenuates a depressive-like phenotype in the FST in healthy animals (Pan et al., 2011). To confirm that CT99021 administration at a concentration of 25 mg/kg delivered by i.p. is a valid treatment in this behavioral paradigm, we injected a solution containing either vehicle or CT99021 and tested animals in the FST 1 h after the injection. As expected, the CT99021-treated group showed significantly reduced immobile times (Figure 2A, 169.0 ± 6.5 in controls and 140.5 ± 6.0 with CT99021 treatment; *p* < 0.01, unpaired *t*-test) demonstrating its validity in behavior tasks and confirmed the effect of CT99021 in the FST.

Using the same testing regime, we examined the effects of CT99021 on basal locomotion and intrinsic anxiety levels using the OFT. The total distance moved in vehicle and CT99021 groups were similar (4,255 ± 225 and 3,893 ± 236 cm, respectively; *p* > 0.05, unpaired *t*-test; Figure 2B), indicating that CT99021 treatment does not affect basal locomotor activity. Vehicle and CT99021 groups spent similar amounts of time in the central region (9.0 ± 1.3% and 7.5 ± 1.0%, respectively; *p* > 0.05, two-way ANOVA; Figure 2C), indicating no difference in intrinsic anxiety levels. In the EPM, there was also no significant difference between vehicle and CT99021 groups, for either time spent in the open (27.0 ± 8.1% and 16.9 ± 5.3%) or closed (59.7 ± 8.1% and 69.4 ± 5.9%) arms, respectively (*p* > 0.05; two-way ANOVA; Figure 2D). Taken together, these results indicate that CT99021 administration results in GSK-3 inhibition in freely moving animals without affecting basal anxiety or gross locomotor function.

### Contextual Fear Memory Is Not Affected by CT99021

Next, we used CFC to investigate hippocampus-dependent contextual fear memory. When tested on the conditioning day, the vehicle and CT99021 groups showed similar levels of freezing (3.0 ± 1.2% and 2.2 ± 0.8%, respectively), which indicates that innate fear to a novel environment is not affected by GSK-3 inhibition. The level of freezing on the retrieval day was also indistinguishable between vehicle and CT99021 groups (40.5 ± 4.1% and 36.3 ± 4.1%, respectively; *p* > 0.05, two-way ANOVA; Figure 2E). In a second set of experiments, foot shock intensity was increased from 0.2 to 0.6 mA. Again, the respective levels of freezing for either vehicle or CT99021 groups on the conditioning day (4.5 ± 1.2% and 5.7 ± 1.4%) and retrieval day (48.2 ± 4.5% and 41.8 ± 3.7%) were similar (*p* > 0.05, two-way ANOVA; Figure 2F).

### CT99021 Enhances Spatial Learning in the Morris Water Maze and T-Maze

**FIGURE 3:**
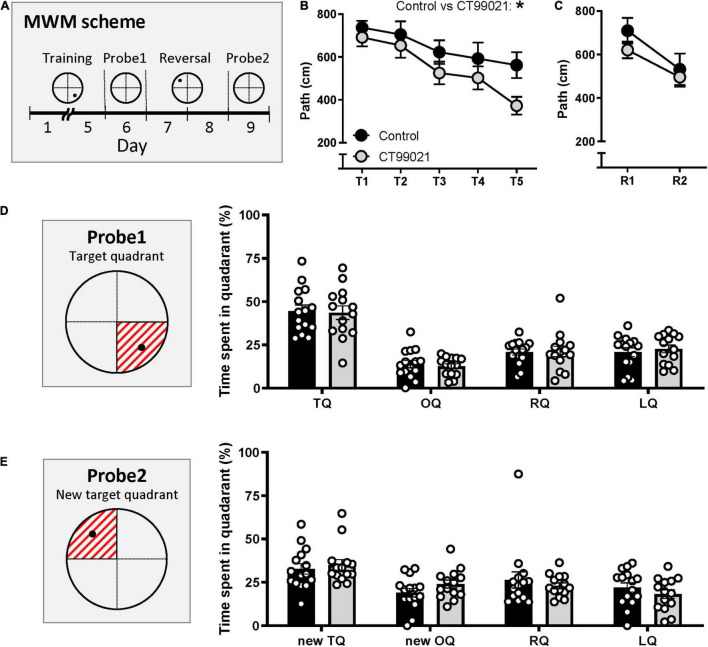
CT99021 improves acquisition in the hidden platform version of the MWM. **(A)** Schematic drawing of the schedule in the MWM. Black circle represents where the hidden platform was placed. **(B)** Path length (cm) to the hidden platform is different between vehicle (*n* = 15) and CT99021 group (*n* = 14) (*p < 0.05, two-way ANOVA). **(C)** Path length to the hidden platform during reversal learning is comparable between two groups (*p* > 0.05, two-way ANOVA). **(D)** (Left) Illustration of the target quadrant represented as the red shaded area on probe 1. (Right) Time spent (%) in four quadrants on probe 1 is not different between control (*n* = 15) and CT99021 group (*n* = 14) (*p* > 0.05, two-way ANOVA). **(E)** (Left) Illustration of the new target quadrant and the time spent in each quadrant on probe 2. (Right) No difference between vehicle and CT99021 groups were observed (*p* > 0.05, two-way ANOVA). Data are presented as mean ± SEM.

Hippocampus-dependent spatial learning was tested in the MWM. During the training sessions (Days 1–5, Figure 3A), the escape latency (s) of the two groups was similar (*p* > 0.05, two-way ANOVA; Supplementary Figure 1A). However, as training continued, the CT99021-treated group took progressively shorter paths to the hidden platform. This trend was consistently observed throughout the training days and resulted in a significant difference between the two groups (*p* < 0.05, two-way ANOVA; Figure 3B). Associated with the reduced path length, there was a trend for the CT99021 group to find the platform more quickly, but swim more slowly (Supplementary Figure 1C).

After five days of training, the first probe test (Day 6, probe 1) was conducted in order to assess long term spatial memory. The time spent in the target quadrant (Figure 3D, left panel) for vehicle and CT99021 groups were comparable (44.6 ± 3.4 s and 43.6 ± 3.9 s, respectively; *p* > 0.05, two-way ANOVA; Figure 3D, right panel).

We also applied Gallagher’s proximity measure, which calculates the cumulative distance from the animal to the hidden platform throughout the session and is a measure of the search error (Gallagher et al., 1993). As expected, the cumulative distance (cm) to the hidden platform during the training days decreased for both groups (Supplementary Figure 2A) and CT99021 improved the performance such that by training day 5 there was a significant difference between the two groups (*p* < 0.05, unpaired *t*-test; Supplementary Figure 2C). There were no differences in the cumulative distances covered in the first probe trial (Supplementary Figure 2D).

Reversal learning was conducted for two days by moving the platform to a different location (Day 7 and 8, Figure 3A). There was no significant difference in the rate of learning of the new location when assessed either using path length measurements (*p* > 0.05, two-way ANOVA; Figure 3C) or escape latency (*p* > 0.05, two-way ANOVA; Supplementary Figure 1B). The swimming speed on reversal was comparable between the two groups (*p* > 0.05, two-way ANOVA, Supplementary Figure 1D). On the second probe day (Day 9, probe 2), CT99021 and vehicle groups stayed in the new target quadrant (Figure 3E, left panel) more than the other quadrants, but no difference was observed between the groups (35.0 ± 3.1 s and 32.7 ± 3.0 s, respectively; *p* > 0.05, two-way ANOVA; Figure 3E, right panel). Cumulative distance from the hidden platfrom during reversal learning and during the second probe trial were also comparable between two groups (Supplementary Figures 2B,E).

**FIGURE 4:**
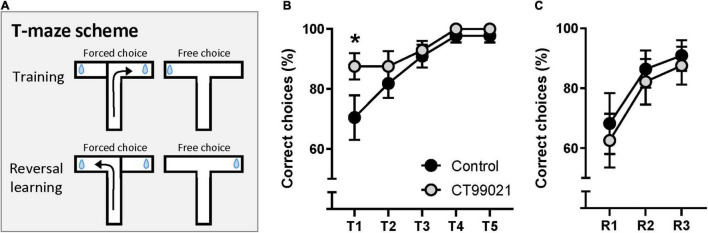
CT99021 improves spatial learning in DMTP T-maze. **(A)** Schematic drawing of DMTP T-maze. The reward arm was switched for reversal learning after five days of training. Blue drop represents a reward. **(B)** Correct choice (%) made by the CT99021 group (*n* = 11) is significantly higher compared to the vehicle group (*n* = 14) on Day 1 (*p < 0.05, two-way ANOVA between vehicle and CT99021; *p < 0.05, Sidak’s *post hoc* test on Day 1). **(C)** No difference between the two groups during reversal learning is observed (*p* > 0.05, two-way ANOVA). Data are presented as mean ± SEM.

For a second test of spatial memory and reversal learning, we conducted a DMTP T-maze. The reward arm was fixed for five training days. This version allows animals to associate the reward with the fixed arm, therefore, success rate was higher than the chance level (50%) on Day 1. The reward arm was switched after Day 5 to assess behavioral flexibility by reversal learning (Figure 4A). On the first day of training, the percentage of correct choices made by the CT99021 group was significantly higher compared to the vehicle group (*p* < 0.05, two-way ANOVA between two groups, 87.5 ± 4.3%, 70.5 ± 7.4%, respectively; *p* < 0.05, on Day 1, Sidak’s *post hoc* test; Figure 4B). Analysis on Day 1 demonstrated that the vehicle group showed gradual improvement in their performance over 4 trials (*p* < 0.05, repeated measures ANOVA, Sidak’s *post hoc* test between trial 1 and 4; Supplementary Figure 3A) while the CT99021 group scored consistently higher during early training (*p* > 0.05, repeated measures ANOVA; Supplementary Figure 3B). This demonstrates that the CT99021 group was more successful at remembering the previously visited arm to choose the alternative arm during the learning period. This superior performance of the CT99021 group was quickly matched by vehicle group and both groups reached mastery by Day 4 (Figure 4B). After Day 5, the reward arm was switched and reversal learning was carried out over three more days. There was no difference in reversal learning performance (*p* > 0.05, two-way ANOVA; Figure 4C) indicating their behavioral flexibility is not affected by inhibition of GSK-3. Taken together, CT99021 improved spatial learning during the active learning period.

### CT99021 Improves Accuracy of Spatial Memory

**FIGURE 5:**
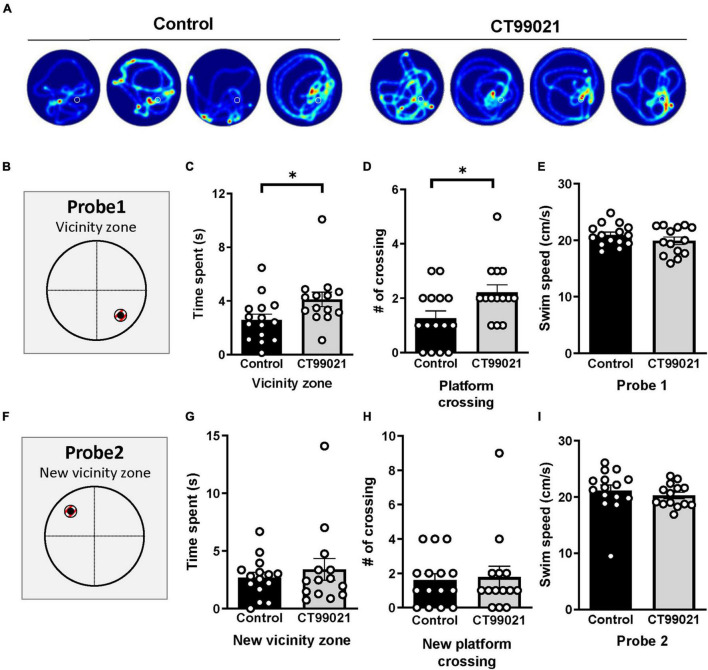
CT99021 improves accuracy of spatial memory. **(A)** Four sample heat map traces on probe 1 from each group are shown. The white circle represents where the hidden platform was placed. **(B)** (Left) Illustration of the vicinity zone for the probe 1 test. **(C)** The CT99021 group showed increased time spent in the vicinity zone (*p < 0.05, unpaired *t*-test). **(D)** Increased number of platform crossings by the CT99021 group (*p < 0.05, unpaired *t*-test). **(E)** Swim speed on probe 1 is indistinguishable between the two groups (*p* > 0.05, unpaired *t*-test). **(F)** The new vicinity zone on the probe 2 trial. **(G)** Time spent in the new vicinity zone is comparable between two groups (*p* > 0.05, unpaired *t*-test). **(H)** The numbers of platform crossings are similar (*p* > 0.05, unpaired *t*-test). **(I)** Swim speed is also comparable on probe 2 (*p* > 0.05, unpaired *t*-test). Control: vehicle group (black histograms), CT99021: drug treatment group (gray histograms). Data are presented as mean ± SEM.

Inspection of the heat map images from the MWM indicated that mice from the CT99021 group spent more time in the vicinity of the platform location (Figure 5A). To quantify this, we applied a zone analysis method (Moser et al., 1993, 1998; de Hoz et al., 2004) narrowing the target area from a quadrant to a small circular zone (Vicinity zone, Figure 5B), which was double the radius of the platform and was centered on the platform location. The vicinity zone covers only 2% of the pool area, and therefore, measures the precision of spatial memory. We found that CT99021-treated mice spent more time in the vicinity zone compared to vehicle-treated mice (4.1 ± 0.5 s and 2.6 ± 0.4 s, respectively; *p* < 0.05, unpaired *t*-test; Figure 5C). Moreover, the number of platform crossings was also increased in the CT99021 group (2.2 ± 0.3 and 1.3 ± 0.3 times, respectively; *p* < 0.05, unpaired *t*-test; Figure 5D). Again, there was no difference in swim speed between groups (19.9 ± 0.7 and 20.9 ± 0.5 cm/s, respectively; *p* > 0.05, unpaired *t*-test; Figure 5E).

On probe 2 after reversal learning (Figure 5F), the CT99021 and vehicle groups were not significantly different in the vicinity zone analysis (3.4 ± 0.9 and 2.7 ± 0.4 s, respectively; *p* > 0.05, unpaired *t*-test; Figure 5G). Similarly, platform crossings (1.8 ± 0.6 and 1.6 ± 0.4 times, respectively; *p* > 0.05, unpaired *t*-test; Figure 5H) and swimming speeds (20.3 ± 0.6 and 21.1 ± 1.0 cm/s, respectively; *p* > 0.05, unpaired *t*-test; Figure 5I) were indistinguishable.

## Discussion

The principal finding of the present study is that a GSK-3 inhibitor, CT99021, blocks the induction of NMDAR-LTD in the CA1 region of the hippocampus *in vivo* and improves hippocampus-dependent learning and memory.

### Effects of CT99021 on Synaptic Function

Here we have found that *in vivo* treatment of CT99021 blocked the induction of hippocampal NMDAR-LTD in adult mice extending previous observations in hippocampal slices prepared from juvenile rats (Peineau et al., 2009). In this previous study, CT99021 and five other structurally distinct GSK-3 inhibitors blocked the induction of NMDAR-LTD, whereas a variety of other inhibitors that targeted many other Ser/Thr kinases, but not GSK-3, were inactive. Like all kinase inhibitors, CT99021 will have off-target effects, but it is the most selective GSK-3 inhibitor known (Bain et al., 2007). While there are other targets of CT99021, CDK2 (cyclin-dependent protein kinase 2), PLK1 (polo like kinase 1) and MELK (maternal embryonic leucine-zipper kinase) (Bain et al., 2007), these proteins are less sensitive than the GSK-3 paralogs to the actions of CT99021 and display little or no expression in the adult CNS (Nguyen et al., 2002; de Carcer et al., 2011; Ganguly et al., 2015). It is most likely, therefore, that the effects observed in the present study are due to inhibition of GSK-3.

The two paralogs of GSK-3, GSK-3α, and GSK-3β, are equi-sensitive to the effects of CT99021, therefore, it is most likely that both paralogs of GSK-3 were inhibited during these experiments. It is unclear the extent to which the actions of CT99021 described here can be attributed to an effect on GSK-3α and/or GSK-3β. Our previous pharmacological studies could not distinguish between the two paralogs. Since LTD was associated with an increase in the activity of the brain-enriched GSK-3β paralog, it was tentatively assumed that GSK-3β was the mediator of LTD (Peineau et al., 2007). Subsequent studies have identified downstream effectors of GSK-3β in LTD, such as PSD-95 (Nelson et al., 2013). However, other more recent work has favored GSK-3α as the paralog responsible for LTD (Shahab et al., 2014; Cymerman et al., 2015; Draffin et al., 2021) or has identified roles for both enzymes (McCamphill et al., 2020). Further studies that, for example, combine pharmacological agents with KO mice will be useful in establishing the relative roles of GSK-3α and GSK-3β in both synaptic plasticity and cognition.

It is possible that other actions of CT99021, rather than its ability to inhibit LTD, are associated with the cognitive effects observed in the present study. In this context, it has been shown that GSK-3 inhibitors reduce the surface expression of synaptic NMDARs (Chen et al., 2017), *via* a mechanism involving GSK-3β-mediated phosphorylation of PI4KIIα (Amici et al., 2020). However, if CT99021 suppressed NMDARs in the present study, we would have expected a reduction in learning and memory in the MWM (Morris et al., 1986) rather than the enhancement we observed. GSK-3β also regulates LTP (Hooper et al., 2007; Liu et al., 2017). Although CT99021 did not affect LTP directly (Jo et al., 2011), it is possible that CT99021 may affect another mechanism that is independent of any action *via* LTD. Whatever the underlying molecular mechanisms are, it is clear that inhibiting GSK-3 paralogs shifts the LTP/LTD balance in favor of LTP. Further studies are warranted to determine the underlying mechanisms by which the GSK-3 paralogs contribute to synaptic plasticity and cognition.

### Effects of CT99021 on Learning and Memory

Previous studies showed that inhibition of GSK-3 by various antagonists in naïve animals does not affect intrinsic anxiety level (Yuskaitis et al., 2010), gross motor function (Watase et al., 2007), hippocampal fear memory (Watase et al., 2007; Miller et al., 2014) and spatial working memory (Tamura et al., 2016). Our observations using CT99021 are in general agreement with these studies.

In the MWM, however, we found that the CT99021-treated group learned the location of the platform more quickly than the vehicle group, as assessed by determining the distance traveled over trials of five successive days. There was a tendency for the swim speed of the CT99021-treated group to be slightly, but consistently, slower during the training period compared to the vehicle-treated group. Since CT99021 does not affect gross motor function observed in the OFT, it is unlikely that this slower speed of the CT99021 group is a reflection of compromised motor function. Considering GSK-3 inhibitors are effective in mouse models of depression (Beaulieu et al., 2009; Jope, 2011; Duda et al., 2020), and have anxiolytic effects in MWM (Nguyen et al., 2017), it is possible that CT99021 is alleviating behavioral stress in the MWM allowing employment of more thoughtful search strategy resulting in improved learning. Consistent with this idea, enhanced LTD has been observed following behavioral stress (Xu et al., 1997; Maggio and Segal, 2009, 2011; Dong et al., 2013). We also observed a faster learning rate with the CT99021-treated group in the DMTP T-maze, where behavioral stress is less likely to be a confound. Considered together, these results suggest that GSK-3 activity impairs the rate of learning.

In the first probe trial, to assess spatial long-term memory, the time in the target quadrant of the two groups was again similar. We noted, however, that the CT99021 group appeared to spend more time in the vicinity of the platform location. To quantify this, we applied a zone analysis method designating an area which covered, but expanded upon, the location where the platform was located previously (Moser et al., 1993, 1998; de Hoz et al., 2004). This variant of the hidden platform task enabled us to assess the accuracy of long term spatial memory. We observed a significant increase in both time spent in this zone and the number of crossings of the platform. This enhancement of spatial memory accuracy further supports the notion that LTD has a role in regulating the precision of memory (Kemp and Manahan-Vaughan, 2007).

In the reversal learning phase of the MWM test, we observed no differences between the two groups, in either the rate of reversal learning or in the memory of the new location. We also observed no effect of CT99021 on reversal learning in the DMTP T-maze. These findings demonstrate that their behavioral flexibility is intact. These results were surprising since previous studies have implicated NMDAR-LTD in behavioral flexibility (Nicholls et al., 2008; Kim et al., 2011, 2017; Dong et al., 2013; Regan et al., 2015). This discrepancy could be for various reasons. In several of these studies (Nicholls et al., 2008; Kim et al., 2011; Regan et al., 2015; Kim et al., 2017) constitutive knockouts were used, and so developmental factors may have introduced confounding variables. The difference between our study and previous pharmacological investigations (Ge et al., 2010; Dong et al., 2013) may relate to the nature of the pharmacological agents used. NMDAR antagonists were used to block *de novo* LTD in behaving mice, spatial memory consolidation (Ge et al., 2010) and behavioral flexibility (Dong et al., 2013). NMDAR antagonists employed in these studies could have blocked other forms of plasticity, for example depotentiation, which has also been suggested to be involved in reversal learning (Zhang and Wang, 2013).

In summary, we have found modest improvements in both learning and memory, which suggests that GSK-3 inhibitors could be mildly cognitive enhancing under normal conditions. The lack of any pronounced effect is in agreement with the general finding that GSK-3 inhibitors have little effect normally (King et al., 2014). In contrast, GSK-3 inhibitors are clearly cognitive enhancers in a wide variety of disease models (King et al., 2014; Duda et al., 2018). One possible explanation for these findings is based upon the LTP/LTD balance hypothesis of cognitive deficits. Under normal conditions, in adult animals, there is little NMDAR-LTD to oppose NMDAR-LTP; the small LTD that we have observed in the present study is consistent with this notion. In contrast, in a variety of disorders, LTD may be enhanced and LTP inhibited such that the suppression of LTD, by inhibition of GSK-3, has more pronounced effects. Consistent with this idea, enhanced LTD has been observed following treatment with toxic oligomeric species of Aβ (Li et al., 2009). The observation that GSK-3 inhibitors are cognitively enhancing in a wide variety of animal models of disease (King et al., 2014; Duda et al., 2018) could be because an alteration in the LTP/LTD balance is a common underlying mechanism in many conditions that affect cognition.

## Data Availability Statement

The original contributions presented in the study are included in the article/Supplementary Material, further inquiries can be directed to the corresponding author/s.

## Ethics Statement

The animal study was reviewed and approved by the Animal Care and Use Committee of Seoul National University and UK Animal Scientific Procedure Act 1986.

## Author Contributions

YL and ZB carried out all the experiments and analyzed the data. YL and GC wrote the manuscript. GC, B-KK, and ZB designed the study and supervised the project. TS, CB, and MZ contributed to the interpretation of the data and the writing of the manuscript. All authors contributed to the article and approved the submitted version.

## Conflict of Interest

The authors declare that the research was conducted in the absence of any commercial or financial relationships that could be construed as a potential conflict of interest.

## Publisher’s Note

All claims expressed in this article are solely those of the authors and do not necessarily represent those of their affiliated organizations, or those of the publisher, the editors and the reviewers. Any product that may be evaluated in this article, or claim that may be made by its manufacturer, is not guaranteed or endorsed by the publisher.
