# Supplementary figures and images for: The GSK-3 Inhibitor CT99021 Enhances the Acquisition of Spatial Learning and the Accuracy of Spatial Memory
Source: Front Mol Neurosci. 2022 Jan 27;14:804130. doi: 10.3389/fnmol.2021.804130 (PMC8829050; doi:10.3389/fnmol.2021.804130)

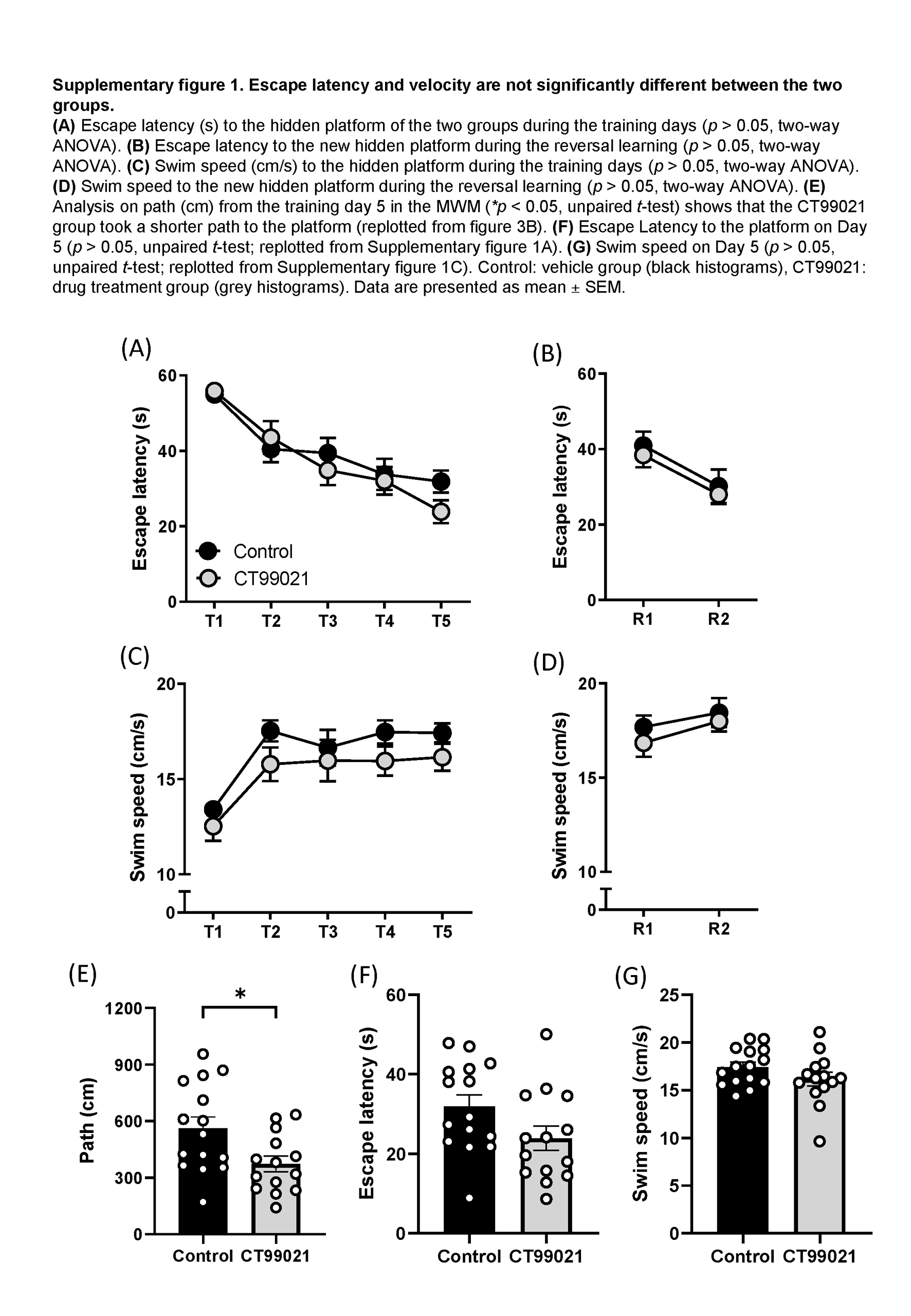

Supplement: Supplementary file 1 [file Image_1.tif]

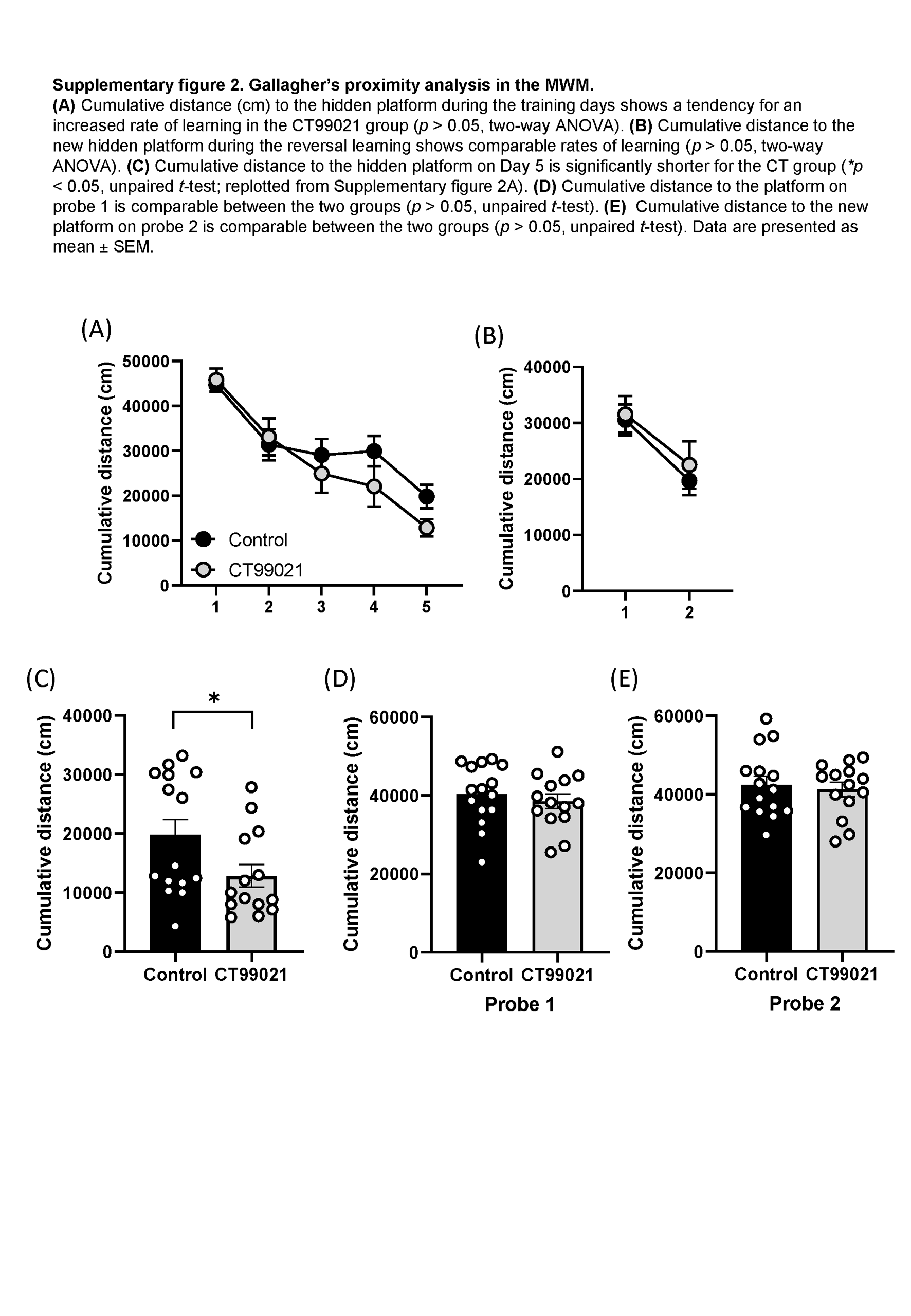

Supplement: Supplementary file 2 [file Image_2.tif]

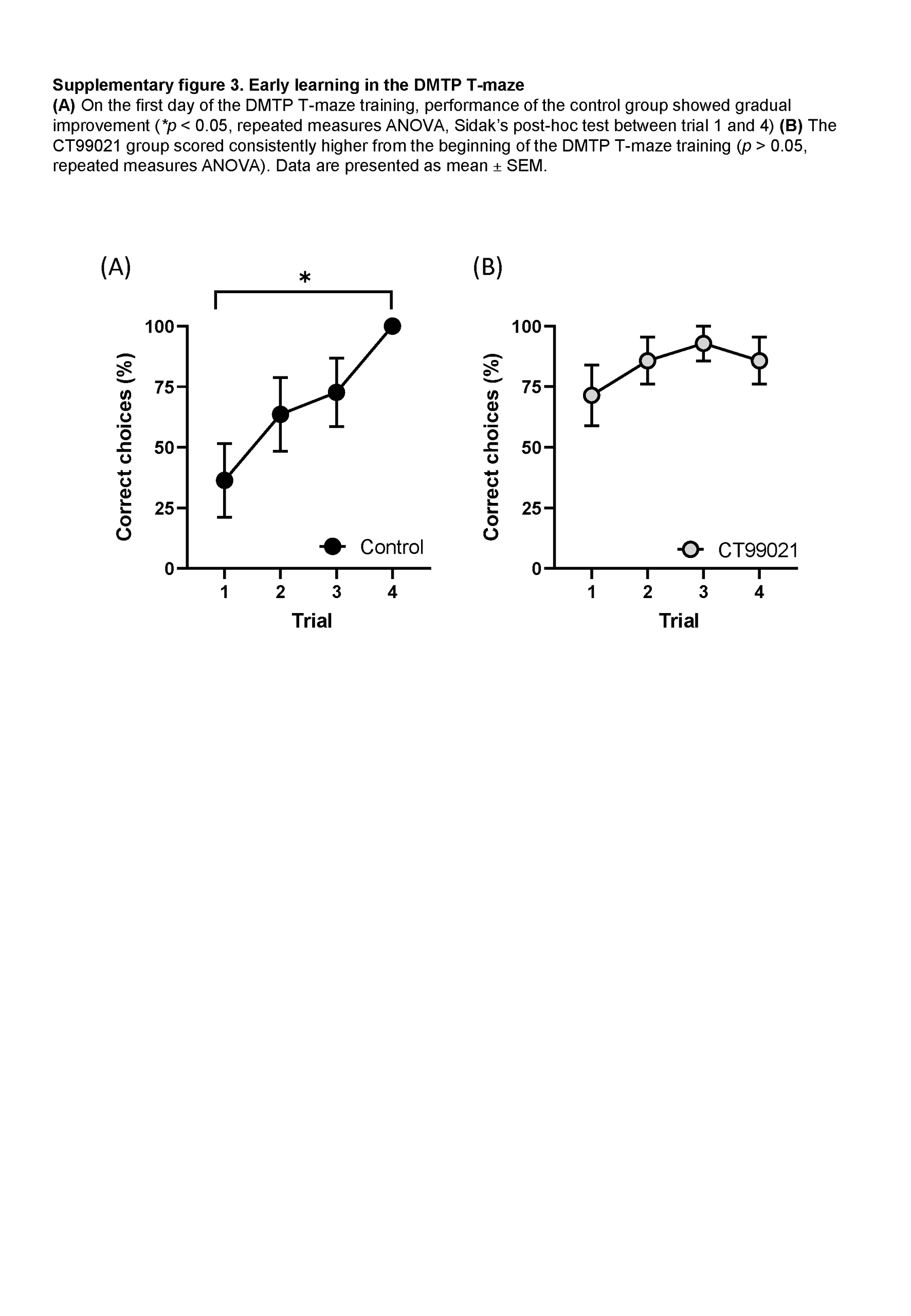

Supplement: Supplementary file 3 [file Image_3.tif]
